# Dose-Response Relationship of Physical Activity to Premature and Total All-Cause and Cardiovascular Disease Mortality in Walkers

**DOI:** 10.1371/journal.pone.0078777

**Published:** 2013-11-29

**Authors:** Paul T. Williams

**Affiliations:** Life Sciences Division, Lawrence Berkeley National Laboratory, Berkeley, California, United States of America; FuWai hospital, Chinese Academy of Medical Sciences, China

## Abstract

**Purpose:**

To assess the dose-response relationships between cause-specific mortality and exercise energy expenditure in a prospective epidemiological cohort of walkers.

**Methods:**

The sample consisted of the 8,436 male and 33,586 female participants of the National Walkers' Health Study. Walking energy expenditure was calculated in metabolic equivalents (METs, 1 MET = 3.5 ml O_2_/kg/min), which were used to divide the cohort into four exercise categories: category 1 (≤1.07 MET-hours/d), category 2 (1.07 to 1.8 MET-hours/d), category 3 (1.8 to 3.6 MET-hours/d), and category 4 (≥3.6 MET-hours/d). Competing risk regression analyses were use to calculate the risk of mortality for categories 2, 3 and 4 relative to category 1.

**Results:**

22.9% of the subjects were in category 1, 16.1% in category 2, 33.3% in category 3, and 27.7% in category 4. There were 2,448 deaths during the 9.6 average years of follow-up. Total mortality was 11.2% lower in category 2 (P = 0.04), 32.4% lower in category 3 (P<10^−12^) and 32.9% lower in category 4 (P = 10^−11^) than in category 1. For underlying causes of death, the respective risk reductions for categories 2, 3 and 4 were 23.6% (P = 0.008), 35.2% (P<10^−5^), and 34.9% (P = 0.0001) for cardiovascular disease mortality; 27.8% (P = 0.18), 20.6% (P = 0.07), and 31.4% (P = 0.009) for ischemic heart disease mortality; and 39.4% (P = 0.18), 63.8% (P = 0.005), and 90.6% (P = 0.002) for diabetes mortality when compared to category 1. For all related mortality (i.e., underlying and contributing causes of death combined), the respective risk reductions for categories 2, 3 and 4 were 18.7% (P = 0.22), 42.5% (P = 0.001), and 57.5% (P = 0.0001) for heart failure; 9.4% (P = 0.56), 44.3% (P = 0.0004), and 33.5% (P = 0.02) for hypertensive diseases; 11.5% (P = 0.38), 41.0% (P<10^−4^), and 35.5% (P = 0.001) for dysrhythmias: and 23.2% (P = 0.13), 45.8% (P = 0.0002), and 41.1% (P = 0.005) for cerebrovascular diseases when compared to category 1.

**Conclusions:**

There are substantial health benefits to exceeding the current exercise guidelines.

## Introduction

The health benefits produced by physical activity are well-documented [1]–[4]. Less is known about the dose-response relationships between specific exercises and mortality. In part, this is because most epidemiological cohorts lack the statistical power to draw inferences for specific activities, and most do not include very many subjects who exercise at high doses. In addition, most studies use exercise duration (i.e., time spent exercising) to calculate the energy expenditure [1], [5]. Our studies of walkers and runners suggest that exercise energy expenditure that is calculated from duration exaggerates the amount exercise performed [6]–[8]. The duration-based estimates also underestimate the health benefits of exercise relative to distance-based estimates (i.e., exercise energy expenditure that is calculated from km run or walked) [6]–[8]. Despite these limitations, studies have shown that walking reduces the risk of total mortality, coronary heart disease, stroke, and diabetes, relative to not walking at all [9]–[27]. However, it is generally not known whether exceeding the recommended dose of physical activity (450 to 750 metabolic equivalents minutes per week [4]) is more beneficial than simply achieving the recommendations. Moreover, even though the relationships between physical activity and disease risks are thought to be nonlinear, formal tests for nonlinearity are generally lacking.

The current report examines the dose-response relationships between exercise energy expenditure and mortality in the National Walkers' Health Study [28]–[30]. Whereas other epidemiological cohorts were designed for general purpose, the National Walkers' Health Study was designed specifically to test the health benefits of walking. Results are presented for all reported exercise, and for walking in particular. In addition, the analyses are not restricted to the underlying cause of death, i.e., “the disease or injury that initiated the chain of morbid events that led directly and inevitably to death” [31]. Contributing causes of death are also included for two reasons: first, a single underlying cause of death may be difficult to identify and may depend upon the physician's training, specialty, and familiarity with the patient [32]–[34]; and second, exercise may affect survival via its effect on one or more of the contributing causes of death.

## Materials and Methods

### Study design

The National Walkers' Health Study is a prospective epidemiological cohort that has been described in detail in several publications [28]–[30]. The cohort was initially recruited between 1998 and 2001 among subscribers of a walking magazine and among participants of walking events. At baseline, participants completed a two-page questionnaire on demographics (age, race, education), walking history (age when began walking at least 12 miles per week, current average weekly mileage), weight history (greatest and current weight; weight when started walking for exercise; least weight as a walker; body circumferences of the chest, waist, and hips; bra cup size), diet (vegetarianism and the current weekly intakes of alcohol, red meat, fish, fruit, vitamin C, vitamin E, and aspirin), current and past cigarette use, history of heart attacks and cancer, and medications for blood pressure, thyroid, cholesterol, or diabetes. The study protocol was approved by the University of California Berkeley Committee for the Protection of Human Subjects, and all subjects provided a signed statement of informed consent.

### Questionnaire items

Intakes of meat, fish and fruit were based on the questions “During an average week, how many servings of beef, lamb, or pork do you eat”, and “…pieces of fruit do you eat”. Alcohol intake was estimated from the corresponding questions for 4-oz. (112 ml) glasses of wine, 12-oz. (336 ml) bottles of beer, and mixed drinks and liqueurs. Alcohol was computed as 10.8 g per 4-oz glass of wine, 13.2 g per 12 oz. bottle of beer and 15.1 g per mixed drink [35]. Correlations between these responses and values obtained from 4-day diet records in 110 men were r = 0.46 and r = 0.38 for consumptions of meat and fruit, respectively. These values agree favorably with published correlations between food records and more extensive food frequency questionnaires for red meat (r = 0.50), and somewhat less favorably for fruit intake (r = 0.50) [36]. Self-reported height and weight from the questionnaire have been found previously to correlate strongly with their clinic measurements (r = 0.96 for both) [24]. Walking energy expenditure was computed by converting the reported distance walked into duration (i.e., reported distance divided by the reported mph speed), which was used in association with reported intensity to calculate MET-hours/d [5]. Energy expenditures from other (non-running and non-walking) activities were calculated from the reported time spent participating in the activities and their published MET values [5].

### Mortality surveillance

Mortality surveillance was completed through December 31, 2008 using the National Death Index (International Classification of Disease codes version 10 [37]). Both underlying cause of death and the entity axis diagnoses of contributing causes were analyzed.

### Statistical analyses

Hazard ratios (HR) from Cox proportional hazard analyses were used to compare all-cause mortality to exercise energy expenditure when adjusted for baseline age (age and age^2^), race, sex, years of education, baseline smoking status (yes/no), ever smoked (yes/no), prior history of a heart attack, prior history of a cancer, aspirin use, and intakes of meat, fruit, and alcohol. Semi-hazard ratios (SHR) from competing risks regression were used to test whether cause-specific deaths were significantly related to exercise energy expenditure when adjusted for these covariates [38]. In these analyses, the competing risk was death due to all other causes. All analyses were performed using the statistical software package Stata (version 11, Stata Corp, College Station, TX). Ninety-five percent confidence intervals (95%CI) are reported for both HR and SHR.

A quadratic expression of MET-hours/d (i.e., MET^2^) was included to test whether the dose-response relationship between mortality and MET-hours/d of exercise was significantly nonlinear. The two- degree of freedom Chi square test was used to test whether men and women showed the same dose-response relationship between mortality and exercise. Specifically, the Chi square test was applied to the difference in the log likelihoods between a model that included separate linear and quadratic regression coefficients for men and women with one that had the same linear and quadratic regression coefficients for both sexes. Results are presented as relative risk (HR, SHR) and their percent reductions in risk (calculated as 100*(HR-1) or 100*(SHR-1)) per MET-hours/d, and for four categories of exercise energy expenditure: category 1 is falling short of the 450 MET minutes per week recommended for health (≤1.07 MET-hours/d), category 2 is meeting the 450 to 750 MET min/wk guideline exercise recommendations (1.07 to 1.8 MET-hours/d [4]), category 3 is exceeding the guidelines by one- to two-fold (1.8 to 3.6 MET-hours/d), and category 4 is exceeding the guidelines by greater than two-fold (≥3.6 MET-hours/d). Access to the data required human use approval.

## Results

Table 1 presents the cohort's baseline characteristics by exercise level. The substantially greater number of females reflects their greater participations in walking events, and their greater proportion among subscribers to walking publications. BMI at baseline (BMI_baseline_) was available for 96.8% of the sample. Greater exercise was associated with younger age, less smoking, fewer prior heart attacks or cancers (females only), less meat and greater fruit and alcohol consumption, and lower BMI_baseline_. BMI when first began walking 12 or more miles per week (BMI_starting walking_) was available for 72.4% of the sample. Most of those with missing BMI_starting walking_ were subjects who simply never achieved that level of activity, i.e. BMI_starting walking_ was unreported for 54.7% of the sample who exercised <1.07 MET-hours/d at baseline, 45.0% of the sample who exercised 1.07 to 1.8 MET-hours/d, 13.2% of the sample who exercised 1.8 to 3.6 MET-hours/d, and 12.6% of the sample who exercised ≥3.6 MET-hours/d.

**Table 1:** Sample characteristics (Mean±SD or percent).

	Total exercise energy expenditure, MET-hours/day:			
	<1.07	1.07–1.8	1.8–3.6	≥3.6
**Females**				
Sample (N)	7591	5456	11232	9307
All exercise (MET-hours/d)¶	0.54±0.31	1.48±0.23	2.66±0.52	6.13±3.09
Walking (MET-hours/d)¶	0.53±0.31	1.42±0.32	2.40±0.75	3.76±1.83
Percent of exercise MET-hours/d due to walking¶	99.09±8.03	96.00±15.81	90.50±22.64	67.40±32.05
Age (y)¶	51.69±14.49	51.27±13.38	50.51±12.62	49.09±12.25
Education (y)§	14.71±2.53	15.05±2.53	15.09±2.51	15.03±2.48
Baseline smoker (%)*	7.38	5.30	4.81	5.17
Ever smoked (%)†	38.16	37.94	39.68	39.92
Prior heart attack (%)§	3.49	3.02	2.06	1.79
Prior cancer (%) §	6.03	5.43	4.62	4.46
Meat (serving/d)¶	0.43±0.41	0.40±0.38	0.37±0.36	0.32±0.33
Fruit (pieces/d)¶	1.32±1.04	1.51±1.08	1.61±1.08	1.75±1.18
Alcohol (g/d)¶	4.24±9.79	5.20±9.85	5.91±10.54	6.14±11.23
BMI_baseline_ (kg/m^2^)¶	28.31±6.68	26.25±5.40	25.26±4.86	24.28±4.42
BMI_starting walking_ (kg/m^2^)¶	27.17±6.81	26.24±5.87	25.77±5.60	25.25±5.59
Mortality (%)¶	6.00%	4.46%	2.78%	2.48%
**Males**				
Sample (N)	2033	1303	2753	2347
All exercise (MET-hours/d)¶	0.54±0.32	1.48±0.22	2.65±0.52	6.41±3.50
Walking (MET-hours/d)¶	0.54±0.32	1.43±0.30	2.43±0.74	3.84±1.92
Percent of exercise MET-hours/d due to walking¶	98.79±9.49	96.81±14.25	91.96±21.78	68.22±34.05
Age (y)§	62.20±14.42	62.07±13.05	61.52±12.41	59.96±12.69
Education (y)	15.60±2.90	15.85±2.83	15.87±2.71	15.90±2.73
Smokers (%)	6.15	4.76	4.29	4.39
Ever smoked (%)	52.53	53.42	54.05	54.07
Prior heart attack (%)†	13.33	12.43	11.44	10.01
Prior cancer (%)	5.95	4.91	5.23	5.28
Meat (serving/d)§	0.51±0.46	0.47±0.44	0.46±0.44	0.43±0.41
Fruit (pieces/d)¶	1.27±1.07	1.44±1.15	1.55±1.19	1.71±1.30
Alcohol (g/d) §	8.95±16.62	10.17±15.88	10.50±16.57	11.41±17.71
BMI_baseline_ (kg/m^2^)¶	28.31±5.52	27.20±4.58	26.99±4.36	26.51±4.17
BMI_starting walking_ (kg/m^2^)†	28.10±6.27	27.21±4.84	27.34±4.98	27.35±5.27
Mortality (%)§	20.22	16.96	12.31	10.31

BMI_baseline_ and BMI when first started walking ≥12 mi/week (BMI_starting walking_) reported by 96.80% and 72.36% of the sample, respectively. Significant of trend by standard linear regression or logistic regression: * P<0.05; † P<0.01; § P<0.0001; ¶ P<10^−15^.

### All-cause mortality

**Figure 1:**
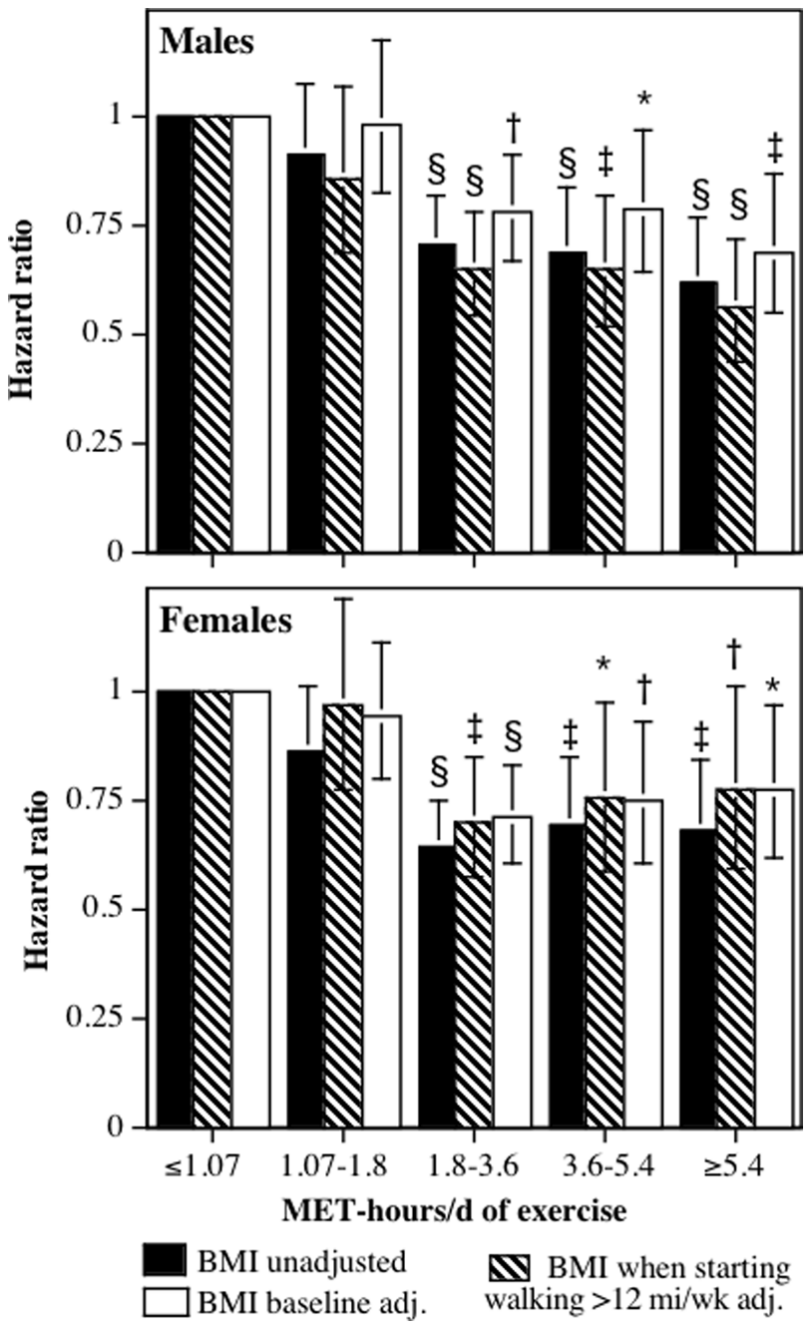
Relative risk (hazard ratio) for all-cause mortality vs. exercise energy expenditure adjusted for age (age plus age^2^), race, education, smoking status (current and prior history), prior history of heart attack and cancer, daily intakes of alcohol, meat and fruit, aspirin use. Brackets designate 95% confidence intervals. Significance levels for the risk reduction relative not achieving the minimum recommended exercise level (i.e., <1.07 MET-hours/d) are coded: * P<0.05, † P<0.01, ‡ P<0.001, and § P<0.0001.

One-thousand two hundred thirteen of the 8,436 men (14.4%) and 1235 of the 33,586 women surveyed at baseline (3.7%) died during the average 9.6-year follow-up. Figure 1 displays the sex-specific reductions in risk associated with greater exercise. Both sexes exhibited significantly greater reductions in risk for exceeding the exercise recommendations compared to simply meeting the recommendations (25% greater risk reduction in males, P = 0.0004; 22.8% greater risk reduction in females, P = 0.001 for ≥1.8 MET-hours/d vs. 1.07 to 1.8 MET-hours/d). Adjustment for medication use and BMI_baseline_ somewhat attenuated the risk reductions. There was no significant difference in the dose-response relationship between males and females (P = 0.93, analyses not displayed), so their data were combined and analyzed adjusted for sex.

When the sexes were combined, Table 2 shows that exercise energy expenditure was inversely related to all cause mortality (significant linear effect), and that the decrease in mortality leveled off (i.e., plateaued) at higher energy expenditures (significant quadratic effect). To depict the degree of nonlinearity the analyses were therefore repeated in Table 3 using the four categories of energy expenditure (see methods). The categorical analysis shows that most of the risk reduction was achieved by 1.8 to 3.6 MET-hours/d. The risk reduction was moderately attenuated by adjustment for baseline medication use and BMI_baseline_ (Table 4) or BMI_starting walking_ (Table 5). Exceeding the exercise recommendations was associated with a 24.1% greater risk reduction compared to merely meeting the recommendations (HR: 0.759, 95%CI: 0.680 to 0.847, P = 10^−6^ for ≥1.8 MET-hours/d vs. 1.07 to 1.8 MET-hours/d).

**Table 2:** Test for significant linear and nonlinear relationships between survival and MET-hours/d of total exercise in walkers.

	Underlying cause of death		All related mortality, i.e., underlying or contributing causes	
	Linear term	Quadratic term	Linear term	Quadratic term
Total mortality*	0.926	1.002		
	(0.907, 0.945)	(1.001, 1.003)		
	P<10^−12^	P = 0.008		
Cardiovascular disease†	0.907	1.003	0.890	1.003
(ICD_10_ I00–I78)	(0.867, 0.948)	(1.002, 1.004)	(0.859, 0.921)	(1.002, 1.004)
	P<10^−4^	P<10^−5^	P<10^−10^	P<10^−13^
Ischemic heart disease†	0.923	1.002	0.907	1.003
(ICD_10_ I20–I25)	(0.867, 0.983)	(1.000, 1.004)	(0.861, 0.956)	(1.001, 1.004)
	P = 0.01	P = 0.05	P = 0.0002	P<10^−4^
Cerebrovascular disease†	0.928	1.002	0.890	1.003
(ICD_10_ I60–I69)	(0.838, 1.028)	(1.000, 1.004)	(0.819, 0.967)	(1.002, 1.005)
	P = 0.15	P = 0.04	P = 0.006	P<10^−4^
Heart failure†	0.834	1.004	0.816	1.005
(ICD_10_ I50)	(0.668, 1.042)	(0.998, 1.010)	(0.738 0.901)	(1.002, 1.007)
	P = 0.11	P = 0.17	P<10^−4^	P<10^−4^
Hypertensive disease†	0.979	1.002	0.884	1.003
(ICD_10_ I10–I13)	(0.809, 1.187)	(0.998, 1.005)	(0.818, 0.956)	(1.002, 1.005)
	P = 0.83	P = 0.35	P = 0.002	P<10^−5^
Dysrhythmia†	0.739	1.006	0.900	1.002
(ICD_10_ I46–I49)	(0.569, 0.961)	(1.002, 1.010)	(0.851, 0.952)	(1.002, 1.004)
	P = 0.02	P = 0.004	P = 0.0003	P<10^−5^
Other cardiovascular disease†	0.975	0.997	0.945	1.000
	(0.805, 1.180)	(0.985, 1.010)	(0.806, 1.109)	(0.987, 1.012)
	P = 0.79	P = 0.65	P = 0.49	P = 0.95
Diabetes†	0.613	1.009	0.700	1.007
(ICD_10_ E10–E14)	(0.448, 0.840)	(1.004, 1.013)	(0.611, 0.802)	(1.005, 1.009)
	P = 0.002	P = 0.0001	P<10^−6^	P<10^−9^

* Hazard ratios (95% confidence intervals) from Cox proportional hazard analyses. † Semi-hazard ratios (95% confidence intervals) from competing risk regression analyses. All analyses adjusted for age (age, age^2^), race, sex, education, prior heart attack, aspirin use, and intakes of red meat, fruit, and alcohol. Total mortality also adjusted for cancer history.

**Table 3:** Survival analyses of underlying cause of death vs. categories of physical activity.

	Total exercise energy expenditure, MET-hours/d			
	1.07 to 1.8	1.8 to 3.6	≥3.6	
Total mortality*	0.888	0.676	0.671	
2448 deaths	(0.792, 0.995)	(0.609, 0.751)	(0.597, 0.753)	
	P = 0.04	P<10^−12^	P<10^−11^	
Cardiovascular disease†	0.764	0.648	0.651	
834 deaths	(0.627, 0.932)	(0.540, 0.777)	(0.530, 0.798)	
	P = 0.008	P<10^−5^	P<10^−4^	
Ischemic heart disease†	0.873	0.686		
443 deaths	(0.664, 1.147)	(0.621, 1.017)	(0.517, 0.910)	
	P = 0.33	P = 0.07	P = 0.009	
Cerebrovascular disease†	0.722	0.693	0.665	
147 deaths	(0.451, 1.158)	(0.457, 1.050)	(0.408, 1.087)	
	P = 0.18	P = 0.08	P = 0.10	
Heart failure†	0.600	0.535	0.578	
53 deaths	(0.274, 1.310)	(0.257, 1.114)	(0.243, 1.373)	
	P = 0.20	P = 0.09	P = 0.21	
Hypertensive disease†	0.408	0.288	0.802	
36 deaths	(0.136, 1.223)	(0.099, 0.841)	(0.319, 2.018)	
	P = 0.11	P = 0.02	P = 0.64	
Dysrhythmia†	1.140	0.347	0.352	
48 deaths	(0.560, 2.318)	(0.149, 0.807)	(0.136, 0.911)	
	P = 0.72	P = 0.01	P = 0.03	
Other circulatory disease†	0.698	0.545	0.794	
107 deaths	(0.400, 1.218)	(0.324, 0.917)	(0.466, 1.352)	
	P = 0.21	P = 0.02	P = 0.39	
Diabetes†	0.606	0.362	0.094	
48 deaths	(0.292, 1.259)	(0.179, 0.732)	(0.022, 0.411)	
	P = 0.18	P = 0.005	P = 0.002	

* Hazard ratios (95% confidence intervals) from Cox proportional hazard analyses. † Semi-hazard ratios (95% confidence intervals) from competing risk regression analyses. See table 2 for adjustments. All hazard ratios relative to <1.07 MET-hours/d.

**Table 4:** Survival analyses of underlying cause of death vs. categories of physical activity adjusted for medication use and BMI_baseline_.

	Total exercise energy expenditure, MET-hours/d		
	1.07 to 1.8	1.8 to 3.6	≥3.6
Total mortality*	0.960	0.743	0.748
	(0.851, 1.084)	(0.665, 0.830)	(0.661, 0.847)
	P = 0.51	P = 10^−6^	P = 10^−5^
			
Cardiovascular disease†	0.871	0.708	0.750
	(0.704, 1.077)	(0.582, 0.862)	(0.602, 0.935)
	P = 0.20	P = 0.0005	P = 0.01
			
Ischemic heart disease†	1.011	0.923	0.877
	(0.752, 1.360)	(0.708, 1.202)	(0.651, 1.182)
	P = 0.94	P = 0.55	P = 0.39
			
Cerebrovascular disease†	0.779	0.655	0.584
	(0.476, 1.274)	(0.418, 1.025)	(0.338, 1.009)
	P = 0.32	P = 0.06	P = 0.05
			
Heart failure†	0.798	0.635	0.681
	(0.329, 1.939)	(0.280, 1.439)	(0.247, 1.877)
	P = 0.62	P = 0.28	P = 0.46
			
Hypertensive disease†	0.462	0.312	0.788
	(0.155, 1.375)	(0.108, 0.899)	(0.306, 2.028)
	P = 0.16	P = 0.03	P = 0.62
			
Dysrhythmia†	1.183	0.381	0.395
	(0.555, 2.522)	(0.161, 0.903)	(0.150, 1.039)
	P = 0.66	P = 0.03	P = 0.06
			
Other circulatory disease†	0.788	0.588	0.959
	(0.436, 1.424)	(0.334, 1.035)	(0.548, 1.677)
	P = 0.43	P = 0.07	P = 0.88
			
Diabetes†	0.791	0.556	0.166
	(0.360, 1.738)	(0.256, 1.207)	(0.038, 0.731)
	P = 0.56	P = 0.14	P = 0.02

* Hazard ratios (95% confidence intervals) from Cox proportional hazard analyses. † Semi-hazard ratios (95% confidence intervals) from competing risk regression analyses. See table 2 for adjustments. Hazard and semi-hazard ratios relative to <1.07 MET-hours/d.

**Table 5:** Survival analyses of underlying cause of death vs. categories of physical activity adjusted for medication use and BMI_starting walking_.

	Total exercise energy expenditure, MET-hours/d		
	1.07 to 1.8	1.8 to 3.6	≥3.6
Total mortality*	0.912	0.672	0.669
	(0.780, 1.067)	(0.588, 0.768)	(0.579, 0.773)
	P = 0.25	P = 10^−8^	P = 10^−7^
Cardiovascular disease†	0.877	0.661	0.679
	(0.671, 1.146)	(0.524, 0.835)	(0.527, 0.875)
	P = 0.34	P = 0.0005	P = 0.003
Ischemic heart disease†	0.927	0.680	0.653
	(0.650, 1.324)	(0.501, 0.924)	(0.467, 0.912)
	P = 0.67	P = 0.01	P = 0.01
Cerebrovascular disease†	0.640	0.783	0.662
	(0.316, 1.298)	(0.453, 1.351)	(0.351, 1.249)
	P = 0.21	P = 0.38	P = 0.20
Heart failure†	1.227	0.890	0.680
	(0.415, 3.628)	(0.337, 2.351)	(0.183, 2.531)
	P = 0.48	P = 0.42	P = 0.57
Hypertensive disease†	0.868	1.570	4.779
	(0.078, 9.698)	(0.228, 10.838)	(0.778, 29.375)
	P = 0.91	P = 0.65	P = 0.09
Dysrhythmia†	1.428	0.340	0.370
	(0.582, 3.501)	(0.133, 0.869)	(0.129, 1.058)
	P = 0.44	P = 0.02	P = 0.06
Other circulatory disease†	0.719	0.600	0.813
	(0.335, 1.543)	(0.315, 1.144)	(0.418, 1.581)
	P = 0.69	P = 0.90	P = 0.59
Diabetes†	0.702	0.411	0.168
	(0.244, 2.023)	(0.164, 1.033)	(0.035, 0.801)
	P = 0.51	P = 0.06	P = 0.03

* Hazard ratios (95% confidence intervals) from Cox proportional hazard analyses. † Semihazard ratios (95% confidence intervals) from competing risk regression analyses. See table 2 for adjustments. Hazard and semihazard ratios relative to <1.07 MET-hours/d.

### Cause-specific mortalities

Sex also did not significantly affect the dose-response relationships between MET-hours/d of exercise and the risk for CVD (P = 0.88 for sex difference), ischemic heart disease (IHD, P = 0.85), dysrhythmias (P = 0.99), heart failure (P = 0.81), hypertensive disease (P = 0.86), cerebrovascular disease (P = 0.89), or diabetes mortality (P = 0.66). Therefore, the male and female data for these deaths were also combined and adjusted for sex when analyzed. Table 2 shows that except for heart failure, the dose-response relationships were all significantly nonlinear. Their analyses by categories of exercise energy expenditure are presented in Tables 3, 4, 5 for underlying causes of death, and in tables 6, 7, 8 for all disease-related mortality (contributing plus underlying cause). Twenty-five percent of IHD-related deaths had a non-CVD underlying cause of death, as did 22% cerebrovascular disease, 31% heart failures, 38% hypertensive disease, and 43% dysrhythmia-related deaths.

**Table 6:** Survival analyses of all related mortality, i.e., underlying or contributing causes of death, vs. categories of physical activity.

	Total exercise energy expenditure, MET-hours/d		
	1.07 to 1.8	1.8 to 3.6	≥3.6
Cardiovascular disease	0.822	0.603	0.611
1320 deaths	(0.704, 0.959)	(0.521, 0.698)	(0.518, 0.720)
	P = 0.01	P<10^−10^	P<10^−8^
Ischemic heart disease	0.768	0.655	0.633
619 deaths	(0.608, 0.969)	(0.532, 0.807)	(0.499, 0.802)
	P = 0.03	P = 0.0001	P = 0.0002
Cerebrovascular disease	0.768	0.542	0.589
254 deaths	(0.545, 1.080)	(0.389, 0.755)	(0.407, 0.853)
	P = 0.13	P = 0.0002	P = 0.005
Heart failure	0.813	0.575	0.425
259 deaths	(0.583, 1.133)	(0.410, 0.807)	(0.274, 0.658)
	P = 0.22	P = 0.001	P = 0.0001
Hypertensive disease	0.906	0.557	0.665
272 deaths	(0.653, 1.257)	(0.401, 0.773)	(0.467, 0.947)
	P = 0.56	P = 0.0004	P = 0.02
Dysrhythmia	0.895	0.590	0.645
498 deaths	(0.701, 1.145)	(0.461, 0.756)	(0.494, 0.843)
	P = 0.38	P<10^−4^	P = 0.001
Other circulatory disease	1.057	1.044	0.788
93 deaths	(0.581, 1.922)	(0.627, 1.739)	(0.430, 1.445)
	P = 0.86	P = 0.87	P = 0.44
Diabetes	0.922	0.398	0.252
191 deaths	(0.646, 1.314)	(0.270, 0.586)	(0.150, 0.423)
	P = 0.65	P<10^−5^	P<10^−6^

Semi-hazard ratios (95% confidence intervals) ratios relative to <1.07 MET-hours/d from competing risk regression analyses. See table 2 for adjustments.

**Table 7:** Survival analyses of all related mortality, i.e., underlying or contributing causes of death, vs. categories of physical activity adjusted for medication use and BMI_baseline_.

	Total exercise energy expenditure, MET-hours/d		
	1.07 to 1.8	1.8 to 3.6	≥3.6
Cardiovascular disease	0.958	0.672	0.707
	(0.814, 1.129)	(0.574, 0.787)	(0.592, 0.844)
	0.61	P<10^−6^	P = 0.0001
Ischemic heart disease	0.907	0.772	0.816
	(0.707, 1.165)	(0.617, 0.968)	(0.635, 1.048)
	P = 0.45	P = 0.02	P = 0.11
Cerebrovascular disease	0.825	0.531	0.610
	(0.575, 1.185)	(0.370, 0.763)	(0.408, 0.914)
	P = 0.30	P = 0.0006	P = 0.02
Heart failure	1.001	0.669	0.498
	(0.700, 1.432)	(0.464, 0.964)	(0.310, 0.800)
	P = 0.99	P = 0.03	P = 0.004
Hypertensive disease	1.126	0.713	0.841
	(0.790, 1.605)	(0.505, 1.006)	(0.573, 1.236)
	P = 0.52	P = 0.05	P = 0.38
Dysrhythmia	1.009	0.636	0.757
	(0.779, 1.306)	(0.489, 0.826)	(0.571, 1.004)
	P = 0.94	P = 0.0007	P = 0.05
Other circulatory disease	1.219	1.284	1.063
	(0.630, 2.358)	(0.715, 2.307)	(0.541, 2.089)
	P = 0.56	P = 0.40	P = 0.86
Diabetes	1.194	0.585	0.407
	(0.807, 1.765)	(0.381, 0.898)	(0.233, 0.712)
	P = 0.37	P = 0.01	P = 0.002

Semi-hazard ratios (95% confidence intervals) ratios relative to <1.07 MET-hours/d from competing risk regression analyses. See table 2 for adjustments.

**Table 8:** Survival analyses of all related mortality, i.e., underlying or contributing causes of death, vs. categories of physical activity adjusted for medication use and BMI_starting walking_.

	Total exercise energy expenditure, MET-hours/d		
	1.07 to 1.8	1.8 to 3.6	≥3.6
Cardiovascular disease	0.857	0.614	0.632
	(0.691, 1.063)	(0.510, 0.740)	(0.516, 0.774)
	P = 0.16	P<10^−6^	P<10^−5^
Ischemic heart disease	0.784	0.596	0.616
	(0.575, 1.071)	(0.460, 0.773)	(0.463, 0.819)
	P = 0.13	P = 0.0001	P = 0.0008
Cerebrovascular disease	0.666	0.622	0.619
	(0.398, 1.114)	(0.407, 0.950)	(0.385, 0.993)
	P = 0.12	P = 0.03	P = 0.05
Heart failure	1.155	0.709	0.549
	(0.727, 1.835)	(0.453, 1.108)	(0.317, 0.951)
	P = 0.54	P = 0.13	P = 0.03
Hypertensive disease	0.992	0.629	0.802
	(0.625, 1.574)	(0.414, 0.955)	(0.517, 1.243)
	P = 0.98	P = 0.03	P = 0.32
Dysrhythmia	0.982	0.573	0.595
	(0.708, 1.362)	(0.422, 0.779)	(0.427, 0.831)
	P = 0.91	P = 0.0003	P = 0.002
Other circulatory disease	1.201	1.203	0.881
	(0.521, 2.769)	(0.606, 2.389)	(0.405, 1.919)
	P = 0.67	P = 0.60	P = 0.75
Diabetes	0.862	0.475	0.347
	(0.502, 1.479)	(0.285, 0.790)	(0.186, 0.645)
	P = 0.59	P = 0.004	P = 0.0008

Semi-hazard ratios (95% confidence intervals) ratios relative to <1.07 MET-hours/d from competing risk regression analyses. See table 2 for adjustments.

### Cardiovascular disease

The risk of CVD as an underlying cause of death decreased 23.6% for meeting the recommended exercise levels, 35.2% for exceeding the recommendations by 1 to 2 fold, and 34.9% for exceeding the recommendations by ≥2-fold (Table 3). Similar results were obtained for all CVD-related deaths (Table 6), which were only moderately attenuated when adjusted for medications and BMI_starting walking_ (Tables 5 and 8). The reduction CVD-related mortality was significantly greater for exceeding the recommendations compared to merely meeting them (SHR: 0.738, 95%CI: 0.634 to 0.859, P = 0.0001). When adjusted for BMI_baseline_ (Tables 4 and 7), the hazard ratios for both 1.8 to 3.6 MET-hours/d and ≥3.6 MET-hours/d remained significant and showed ≥25% risk reductions relative to inadequate exercise.

### Ischemic heart disease

IHD represented about one-half of all CVD deaths. The reduction in risk for all IHD-related mortality was similar to that of all CVD-related mortality (Table 6). Adjustment for baseline medication use and BMI_starting walking_ did not weaken the reductions in risk (Table 8), whereas adjustment for BMI_baseline_ did (Table 7). Nevertheless, exceeding the recommendations by ≥1.8 MET-hours/d was associated with a 21.1% risk reduction relative to inadequate exercise for all IHD-related deaths, even when adjusted for BMI_baseline_ (SHR: 0.789, 95%CI: 0.647 to 0.963, P = 0.02).

### Cerebrovascular disease

The risk for death due to cerebrovascular disease as an underlying cause was significantly less in those who exercised ≥1.8 MET-hours/d vs. <1.07 MET-hours/d (SHR: 0.682, 95%CI: 0.470 to 0.989, P = 0.04), which persisted when adjusted for medication use and BMI_baseline_ (SHR: 0.627, 95%CI 0.417 to 0.942, P = 0.03). The total number of events was increased substantially by including deaths with cerebrovascular disease listed as a contributing cause. Tables 6, 7, 8 show that the risk for all cerebrovascular disease-related deaths decreased significantly in association with exercising 1.8 to 3.6 and ≥3.6 MET-hours/d vs. inadequate exercise, with or without adjustment for BMI (BMI_baseline_ or BMI_starting walking_). Two-thirds of the diagnoses were “stroke not specified as hemorrhage or infarction” or “unspecified cerebrovascular disease” that prevented a more refined endpoint analyses.

### Dysrhythmias

Despite only 48 deaths ascribed to dysrhythmias as the underlying cause, their risk was significantly less in those who exercised ≥1.8 MET-hours/d vs. <1.07 MET-hours/d (Table 3), which generally persisted when adjusted for baseline medication use, BMI_baseline_ (Table 4), or BMI_starting walking_ (Table 5). There was little difference in the risk reduction for 1.8 to 3.6 MET-hours/d and ≥3.6 MET-hours/d. The risk for dysrhythmia as an underlying cause of death was 60% to 65% lower for ≥1.8 vs. <1.07 MET-hours/d without any BMI adjustment (SHR: 0.349, 95%CI: 0.170 to 0.717, P = 0.004), when adjusted for BMI_starting walking_ (SHR: 0.352, 95%CI: 0.151 to 0.814, P = 0.02), and when adjusted for BMI_baseline_ (SHR: 0.386, 95%CI: 0.183 to 0.814, P = 0.01). There were ten times as many dysrhythmia-related deaths (underlying and contributing) than dysrhythmias as an underlying cause. The risk for dysrhythmia-related deaths was significantly lower for 1.8 to 3.6 MET-hours/d and ≥3.6 MET-hours/d vis-à-vis <1.07 MET-hours/d, with or without BMI adjustment.

### Heart failure

There were only 53 deaths due to heart failure as an underlying cause, but five times as many heart failure-related deaths, and their risk was significantly reduced by exercising 1.8 to 3.6 MET-hours/d and ≥3.6 MET-hours/d vis-à-vis <1.07 MET-hours/d, with or without BMI adjustment (Tables 6–8). Exceeding the exercise recommendations reduced the risk of heart failure by 36.6% (SHR: 0.634, 95%CI: 0.444 to 0.906, P = 0.01) relative to simply meeting the recommendations.

### Other circulatory diseases

Deaths due to other circulatory diseases (i.e., exclusive of IHD, cerebrovascular disease, heart failure, dysrhythmia, or hypertensive disease) were unrelated to physical activity.

### Diabetes

The risk for diabetes as an underlying cause of death was about two-thirds lower for 1.8 to 3.6 MET/d, and 90% lower for ≥3.6 MET/h vis-à-vis inadequate exercise, with the reduction in risk for ≥3.6 MET-hours/d remaining significant when adjusted for BMI. The larger number of all diabetes-related deaths strengthen the significance of the risk reduction for both 1.8 to 3.6 MET/d and ≥3.6 MET/h vs. inadequate exercise, with or with BMI adjustment (Tables 6–8).

### Premature (age<65) mortality

There were 467 deaths before age 65, including 165 CVD-related, 66 dysrhythmia-related, 63 IHD-related, 35 hypertensive disease-related, 14 heart failure-related- and 35 diabetes-related deaths. No one died from a cerebrovascular disease-related death before the age of 65. Relative to exercising inadequately, expending between 1.07 to 1.8 MET-hours/d, 1.8 to 3.6 MET-hours/d, and ≥3.6 MET-hours/d was associated with: 1) 25.4% (P = 0.04), 27.3% (P = 0.005), and 29.7% (P = 0.01) lower risk for all-cause premature mortality, respectively; 2) 11.7% (P = 0.58), 34.0% (P = 0.04), and 38.6% (P = 0.03) lower risk for all premature CVD-related mortality, respectively; 3) 47.7% (P = 0.58), 44.2% (P = 0.04), and 49.8% (P = 0.03) lower risk for all premature IHD-related mortality, respectively; 4) 22.9% (P = 0.45), 59.8% (P = 0.007), and 52.4% (P = 0.03) lower risk for all premature dysrhythmia-related mortality, respectively; 5) 33.1% (P = 0.38), 53.9% (P = 0.08), and 36.6% (P = 0.03) lower risk for all premature hypertensive disease-related mortality, respectively; 6) 65.1% (P = 0.20), 83.2% (P = 0.03), and 89.8% (P = 0.02) lower risk for all premature heart failure-related mortality, respectively; and 7) 52.8% (P = 0.14), 60.3% (P = 0.04), and 76.1% (P = 0.005) lower risk for all premature diabetes-related mortality, respectively.

### Excluding early mortality

All of the preceding analyses excluded mortality within the first year of follow-up. Excluding deaths during the first three years of follow-up left 2029 total deaths, including 1084 CVD-related, 494 IHD-related, 414 dysrhythmia-related, 231 hypertensive disease-related, 216 heart failure-related, 207 cerebrovascular disease-related, and 160 diabetes-related deaths. Relative to exercising inadequately, expending between 1.07 to 1.8 MET-hours/d, 1.8 to 3.6, MET-hours/d and ≥3.6 MET-hours/d were associated with: 1) 6.0% (P = 0.32), 27.2% (P = 10^−7^), and 16.5% (P = 0.01) lower risk for total mortality, respectively; 2) 12.8% (P = 0.12), 37.3% (P = 10^−8^), and 31.8% (P = 10^−5^) lower risk for all CVD-related mortality, respectively; 3) 16.4% (P = 0.17), 30.7% (P = 0.002), and 29.5% (P = 0.009) lower risk for all IHD-related mortality, respectively; 4) 7.0% (P = 0.60), 40.2% (P = 0.0001), and 31.6% (P = 0.01) lower risk for all dysrhythmia-related mortality, respectively; 5) 1.7% (increase, P = 0.92), 43.3% (P = 0.002), and 25.2% (P = 0.13) lower risk for all hypertensive disease-related mortality, respectively; 6) 21.0% (P = 0.22), 45.7% (P = 0.0006), and 31.3% (P = 0.07) lower risk for all cerebrovascular disease-related mortality, respectively; 7) 14.8% (P = 0.39), 39.6% (P = 0.007), 55.0% (P = 0.0004) lower risk for all heart failure-related mortality, respectively; and 8) 11.2% (P = 0.55), 59.7% (P = 10^−6^), and 76.9% (P = 10^−6^) lower risk for all diabetes-related mortality, respectively.

### Walking

**Figure 2:**
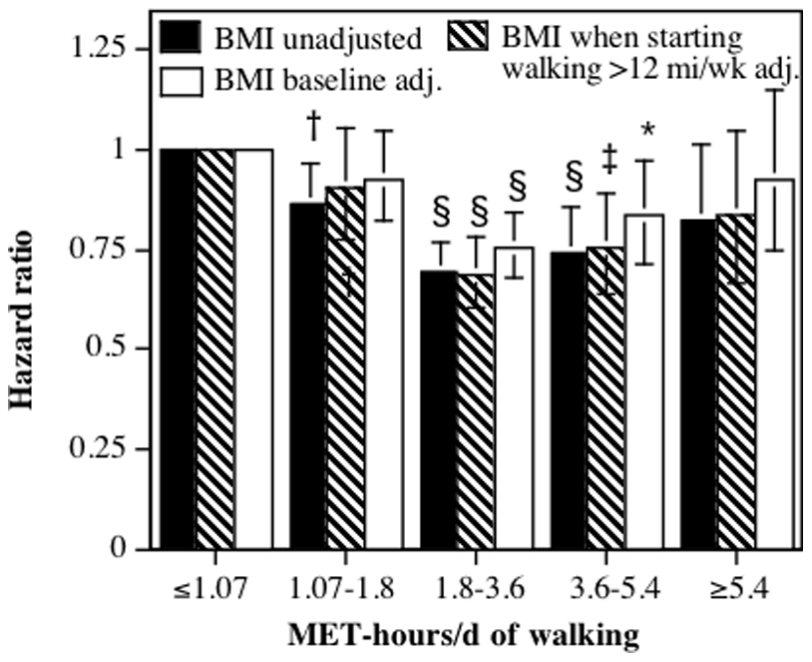
Relative risk (hazard ratio) for all-cause mortality vs. walking energy expenditure adjusted for sex, age (age plus age^2^), race, education, smoking status (current and prior history), prior history of heart attack and cancer, daily intakes of alcohol, meat and fruit, aspirin use. Brackets designate 95% confidence intervals. Significance levels for the risk reduction relative not achieving the minimum recommended exercise level (i.e., <1.07 MET-hours/d) are coded: * P<0.05, † P<0.01, ‡ P<0.001, and § P<0.0001.

Walking represented more than 96% of the exercise energy expenditure <1.8 MET-hours/d, 90% to 92% of the expenditure between 1.8 and 3.6 MET-hours/d, but only 67% to 68% of energy expenditure above 3.6 MET-hours/d. The proportions were very similar for both males and females (Table 1). Not surprisingly, then, the results for walking are very similar to those for total exercise, particularly at the lower range of energy expenditure. Figure 2 show that the decrease in risk was greatest between 1.8 and 3.6 MET-hours/d, and although the risk reduction diminished at higher levels of walking energy expenditure, there was no significant difference in the risk reductions above 1.8 MET-hours/d. Only 5% of the sample expended ≥5.4 MET-hours/d. Table 9 shows that generally, the reductions in risk associated with walking energy expenditure were very similar, albeit slightly weaker in magnitude, than those observed for all exercise.

**Table 9:** Survival analyses of all related mortality, i.e., underlying or contributing causes of death vs. categories of walking energy expenditure.

	Walking energy expenditure, MET-hours/d		
	1.07 to 1.8	1.8 to 3.6	≥3.6
Total mortality*	0.866	0.693	0.764
	(0.774, 0.968)	(0.627, 0.767)	(0.672, 0.868)
	P = 0.01	P<10^−12^	P<0.0001
Cardiovascular disease†	0.817	0.645	0.651
	(0.702, 0.951)	(0.560, 0.742)	(0.540, 0.784)
	P = 0.009	P = 10^−9^	P<10^−5^
Ischemic heart disease†	0.740	0.693	0.718
	(0.587, 0.933)	(0.566, 0.848)	(0.551, 0.937)
	P = 0.01	P = 0.0004	P = 0.02
Cerebrovascular disease†	0.726	0.621	0.624
	(0.514, 1.026)	(0.453, 0.851)	(0.408, 0.954)
	P = 0.07	P = 0.003	P = 0.03
Heart failure†	0.852	0.553	0.591
	(0.615, 1.179)	(0.393, 0.779)	(0.372, 0.941)
	P = 0.33	P = 0.007	P = 0.03
Hypertensive disease†	0.891	0.683	0.763
	(0.642, 1.238)	(0.502, 0.929)	(0.515, 1.132)
	P = 0.49	P = 0.02	P = 0.18
Dysrhythmia†	0.893	0.596	0.674
	(0.702, 1.134)	(0.470, 0.757)	(0.498, 0.912)
	P = 0.35	P = 10^−4^	P = 0.01
Other circulatory disease†	1.085	0.973	0.599
	(0.619, 1.900)	(0.593, 1.597)	(0.290, 1.236)
	P = 0.77	P = 0.92	P = 0.17
Diabetes†	0.844	0.440	0.363
	(0.584, 1.218)	(0.302, 0.641)	(0.210, 0.627)
	P = 0.36	P = 10^−4^	P = 0.0003

* Hazard ratios (95% confidence intervals) from Cox proportional hazard analyses. † Semi-hazard ratios (95% confidence intervals) from competing risk regression analyses. See table 2 for adjustments. Hazard and semi-hazard ratios relative to <1.07 MET-hours/d.

## Discussion

The National Walkers' Health Study was specifically recruited to characterize the dose-response relationships between walking and health. Its highest walking category, i.e. ≥5.4 MET-hours/d, corresponds to ≥1.4 hours or ≥5.0 miles (8 km) per day of brisk walking. This exercise category is substantially greater than those included in other studies. It is greater than all but one of the studies included in Hamer and Chida's meta-analyses of CVD [16], all of those included in Jeon et al.’s meta-analyses of diabetes [27], and all of those included in Moose et al meta-analyses of over 650,000 subjects [39]. It is over twice as great as the top walking categories reported by Manson et al. for the Nurses' Health Study [9] and for the Women's Health Initiative Observational Study [10]. The inclusion of highly active walkers in our study allows us to conclude that most of the reductions in total, CVD, and diabetes mortality appears to be achieved by exercising between 1.8 and 3.6 MET-hour/d.

### All cause mortality

Consistent with prior studies [17], [18], [21]–[23], [39] our analyses showed that greater exercise was associated with lower all-cause mortality. However, they also showed significantly greater risk reductions for patients who exceeded the exercise recommendations relative to simply achieving them, with additional benefits accruing through at least 2.7 MET-hours/d (the midpoint and average of the 1.8 to 3.6 MET-hour/d category). Moreover, reductions in all-cause mortality were demonstrated for both total and premature deaths, in both men and women, and excluding deaths during the first three years of follow-up. The 2008 physical activity guidelines suggested a target of 2 to 2.5 h/wk of moderate intensity activity for reducing total mortality [1]. Our results suggest that this would provide only about one-half of the potential risk reduction, and that 2.7 MET-hours/d (5 h or 28 km/wk) is a better target. The 32.9% risk reduction we observed is not inconsistent with the 40% risk reduction for 7 hr/wk presented in the 2008 recommendations [1], and the approximately 35% risk reduction suggested by the analyses of Moore et al. [39].

### Cardiovascular disease

Table 3 shows that significant risk reductions for all CVD and IHD were achieved by meeting the recommendations, with significant further reductions in all CVD mortality by exceeding them. Our data showed no additional risk reduction beyond twice the recommended level. For both diagnoses, the nonlinearity of the dose-response relationship was statistically significant. In addition, our data suggested that the reductions in the risks for hypertensive disease, heart failure, dysrhythmias, and other CVD as underlying causes of death were as great, or greater, than that of IHD. Others also report that the reductions in CVD risk with walking are not limited to IHD [23]. As with IHD, the dose-response relationships for these other conditions were all significantly nonlinear, with the majority of their risk reductions occurring by 1.8 to 3.6 MET-hours/d (Table 2). The 30% to 40% reductions in cerebrovascular disease mortality we observed were somewhat greater than the 25% to 30% reductions reported for physical activity by others [1], perhaps reflecting our use of the distance-based metric. Remarkably, there were five mutually exclusive CVD endpoints (i.e., underlying causes) that showed consistently lower mortality with greater MET-hours/d walked.

### Underlying vs. contributing causes of death

Unlike other studies, the current analyses were not restricted to the underlying cause of death. There is no evidence from Tables 2 to 8 that restricting the analyses to the underlying cause of death was more informative than including all related deaths. In other words, there is no evidence that the reductions in risk were diluted by including contributing causes of death for some other underlying cause. In fact, exercise was associated with lower CVD risk even when CVD was not the underlying cause. This means that the mechanism by which walking reduces mortality may not simply involve the underlying cause, but also conditions linking the initial underlying cause to death. We also caution that the assignment of an underlying cause of death by physicians may be problematic [32]–[34] and that our analyses of all listed causes may circumvent the difficulty of choosing a single underlying cause among several viable alternatives.

### Underestimating the health benefits of walking

Our cohort did not include a totally sedentary group. Therefore, the risk reductions reported here probably underestimate the potential for risk reduction relative to completely sedentary populations. Specifically, others report substantial risk differences that fall within our lowest exercise category (0 to 1.07 MET-hours/d). For example, the Women's Health Study reported that there was a 63% difference in coronary heart disease risk and 44% difference in diabetes risk between non-walkers and those walking between 1 and 1.5 h/wk [11], [26]. In addition, the Nurses' Health Study [9] and the Women's Health Initiative Observational [10] reported that most of reduction in cardiovascular disease and ischemic heart disease risk had occurred by the 70^th^ percentile of their walking distribution. Similarly, the Health Professionals' Follow-up Study reported that there was a 40% reduction in total mortality by the 60^th^ percentile of their walking distribution [17].

### Comparison with runners

The current findings for walkers differ somewhat from our prior analyses of runners, in which we showed prospectively that the risk for nonfatal coronary heart disease decreased 30% (P = 0.006) by running ≥6 MET-hours/d when compared to running 3–6 MET-hours/d [40]. In addition, men and women who ran ≥4.1 MET-hours/d were at significantly lower risk for stroke than those running 2 to 4 MET-hours/d, and those that ran ≥8.2 MET-hours/d were at 60% lower risk than those who ran <2 MET-hours/d [41]. In contrast, Table 3 suggests that the majority of the risk reduction for IHD and cerebrovascular disease occurred by 1.8 to 3.6 MET-hours/d of walking. Several factors may explain the difference: 1) vigorous exercise (e.g. running) may produce different health benefits than moderate intensity exercise (e.g., walking); 2) runners may differ genetically or behaviorally from walkers; and 3) exercise may affect nonfatal and fatal coronary heart disease differently.

### Adjustment for BMI and diabetes, blood pressure, and cholesterol medication use

Tables 4 and 7 showed that the risk reduction for most causes of death with exercise (CVD-, IHD-, cerebrovascular disease-, heart failure-, dysrhythmia-, and diabetes-related deaths) remained significant when adjusted for BMI_baseline_ and medication use. The reductions in risk were somewhat diminished by the adjustment, but nevertheless remained clinically and statistically very significant. These analyses likely represent over-adjustment because running and walking are known to reduce hypertension, high cholesterol and type 2 diabetes risk [42]–[45], and to attenuate age-related weight gain [46], [47]. In this regard, the adjustments for BMI_starting walking_ in Tables 5 and 8 are probably more appropriate than the adjustment for BMI_baseline_, given that weight control may mediate some of the health benefits of walking.

### Limitations

There are important limitations to these analyses. Vital status in known only from the National Death Index and therefore some subjects who have died are likely to be misclassified as alive. The set of covariates used in the analyses are somewhat restricted and all are measured with error, which will cause their effect to be underestimated by the adjustment. The ability to exercise harder may be an innate quality of health and not directly related to habitual physical activity. The greater risk for heart failure mortality with less exercise could be a consequence of cardiac insufficiency at an early, preclinical, stage of the disease.

### Conclusions

These analyses suggest there are important health benefits to exceeding the current exercise recommendations for health (≥750 MET minutes per week or ≥1.8 MET-hours/d [9]) rather than just satisfying them (450 to 750 MET minutes per week [9]). These health benefits include reductions in disease mortality that are not traditionally associated with walking, including both heart failure and dysrhythmias.
